# Highly Sensitive Twin Resonance Coupling Refractive Index Sensor Based on Gold- and MgF_2_-Coated Nano Metal Films

**DOI:** 10.3390/bios11040104

**Published:** 2021-04-02

**Authors:** Kawsar Ahmed, Mohammed A. AlZain, Hasan Abdullah, Yanhua Luo, Dhasarathan Vigneswaran, Osama S. Faragallah, Mahmoud M. A. Eid, Ahmed Nabih Zaki Rashed

**Affiliations:** 1 Group of Biophotomatiχ, Department of ICT, MBSTU, Tangail 1902, Bangladesh; hasanabdullah989@gmail.com; 2 Department of Information Technology, College of Computers and Information Technology, Taif University, P.O. Box 11099, Taif 21944, Saudi Arabia; m.alzain@tu.edu.sa (M.A.A.); o.salah@tu.edu.sa (O.S.F.); 3 Photonics & Optical Communication, School of Electrical Engineering & Telecommunications, University of New South Wales, Sydney, NSW 2052, Australia; yanhua.luo1@unsw.edu.au; 4 Department of Electronics and Communication, Sri Krishna College of Technology, Coimbatore 641 042, India; dhasa.viki@gmail.com; 5 Department of Electrical Engineering, College of Engineering, Taif University, P.O. Box 11099, Taif 21944, Saudi Arabia; m.elfateh@tu.edu.sa; 6 Department of EECE, Faculty of Electronic Engineering, Menoufia University, Menouf 32951, Egypt; ahmed.rashed@el-eng.menofia.edu.eg

**Keywords:** birefringence, PCF, refractive index sensor, surface plasmon resonance, sensitivity

## Abstract

A plasmonic material-coated circular-shaped photonic crystal fiber (C-PCF) sensor based on surface plasmon resonance (SPR) is proposed to explore the optical guiding performance of the refractive index (RI) sensing at 1.7–3.7 μm. A twin resonance coupling profile is observed by selectively infiltrating liquid using finite element method (FEM). A nano-ring gold layer with a magnesium fluoride (MgF${}_{2}$) coating and fused silica are used as plasmonic and base material, respectively, that help to achieve maximum sensing performance. RI analytes are highly sensitive to SPR and are injected into the outmost air holes of the cladding. The highest sensitivity of 27,958.49 nm/RIU, birefringence of 3.9 × $10^{-4}$, resolution of 3.70094 × $10^{-5}$ RIU, and transmittance dip of −34 dB are achieved. The proposed work is a purely numerical simulation with proper optimization. The value of optimization has been referred to with an experimental tolerance value, but at the same time it has been ensured that it is not fabricated and tested. In summary, the explored C-PCF can widely be eligible for RI-based sensing applications for its excellent performance, which makes it a solid candidate for next generation biosensing applications.

## 1. Introduction

PCF has gained widespread attention among scientists and researchers because of its exceptional amenities facilities over conventional fibers, such as high birefringence [1], broad modal area [2], tunable nonlinearity [3], endless single mode [4], very low confinement loss [5], and tunable dispersion [6,7]. In recent years, SPR is one of the distinguished sensing technology that is applied in the large border area of sensing application fields [8,9,10,11]. In metal-coated PCF, SPR is formed when the phase-matching condition is fulfilled, which means that the surface plasmon polarization (SPP) mode and fundamental mode belong to the same propagation constant at a specific wavelength [12,13,14,15]. The surface plasmon effect is one of the excellent characteristics of metal-coated PCF, which is broadly used for chemical sensing [16], gas sensing [17], bio-sensing [18], pressure sensing [19,20], metamaterial absorption [21,22,23], biochemical reaction measuring [24], and solar energy absorption [25,26].

In recent years, various materials, such as gold, copper, silver, zinc, and aluminum, have been used in PCF structures to generate a plasmonic effect, which plays a significant role in RI sensing applications [27,28]. In 2016, Gangwar et al. described an SPR RI sensor based on PCF and attained a sensitivity response of 7700 nm/RIU and amplitude resolution of 1.3 × $10^{-5}$ [29]. Though they obtained high amplitude resolution, the sensitivity response was very low in the small RI analyte range. In the next year, an SPR sensor is displayed by An et al. to improve the sensitivity response of 10,493 nm/RIU at 1.38 using a gold layer [30]. They improved the sensitivity, but the operating RI analyte range (1.33–1.38) was not high enough. In 2018, Rifat et al. explored a plasmonic sensor with a sensitivity response of 11,000 nm/RIU and amplitude resolution of 9.1 × $10^{-6}$, but the wavelength at which they operated was very low [31]. In 2020, Nan Chen et al. proposed a D-shaped gold layer-based PCF for the detection RI ranges from 1.36 to 1.37 within the wavelength rages from 2.9 μm to 3.6 μm where this structure achieved a maximum sensitivity of 11,500 nm/RIU [32]. Their obtained sensitivity response and RI range were not good enough. In the same year, Yongbo et al. proposed an SPR-based gold-coated PCF with a sensitivity resolution of 2.12 × $10^{4}$ nm/RIU and 4.72 × $10^{-6}$, respectively [33]. Despite this improved sensitivity response, the RI range was very low. Earlier in 2020, a D-shaped double loss peak-based SPR sensor is proposed and achieved maximum sensitivity of 18,900 nm/RIU [34]. From the background studies, it is clear that twin-resonance-based RI sensors with high sensitivity performance are very limited. Although the researchers have obtained good sensitivity, it can be said from the literature studies that there are numerous opportunities to design a biosensor to gain a good sensitivity profile with a border RI range of analytes.

In this work, a twin-resonance RI sensor based on SPR is proposed using the FEM method. A nanofilm gold–MgF${}_{2}$ composite layer is used as a plasmonic material to obtain maximum sensing performance. A simple PCF structure is designed that provides better sensing performance than the previous reports. The background material is fused silica and highly SPR-sensitive RI analytes are injected into the outmost air holes of the cladding. The proposed C-PCF exhibition may be broadly favorable for RI sensing areas due to its prominent sensing profile.

## 2. Structural Design and Methodology

**Figure 1:**
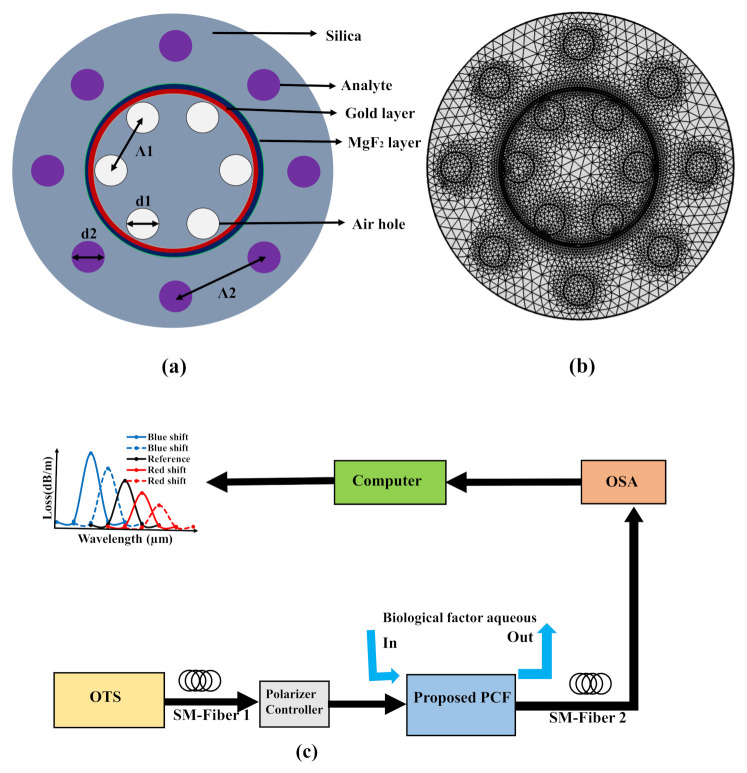
The (**a**) cross-sectional area, (**b**) mesh diagram and (**c**) schematic model of the proposed C-PCF.

A schematic diagram, cross-sectional view, and mesh analysis of the proposed C-PCF are demonstrated in Figure 1. In the simulation process, a circular perfectly match layer (C-PML) is used as boundary condition with thickness of 10% of the radius of the proposed sensor for efficient calculation of loss profile. In this structure, the finite element mesh (FEM) and scattering boundary condition are used in the calculation process to discover the modal properties. The FEM divides the proposed sensor into homogeneous subspaces which are either triangular or quadrilateral in shape. The neighboring subspaces promote solving Maxwell’s equations using FEM. This FEM also helps obtaining the mode field pattern and effective index with more accurate results. Furthermore, the number of elements, boundary elements, and vertex elements as 27,978, 4071, and 578, respectively, are founded applying FEM. In addition, the FEM boosts receiving the minimum element quality of about 0.8127. For the SPR excitation, a gold layer and MgF${}_{2}$ layer were used in the structure. As a dielectric, MgF${}_{2}$ is thoroughly used for its high stability and metallic purities. The dielectric layer on the metal film can prevent corrosion. The gold layer is represented in red, and the MgF${}_{2}$ layer in dark blue. One-layer circular air holes, indicated in white, are circularly inside the gold layer ring, with diameter d1 = 1 μm and lattice pitch Λ1 = 3.950 μm. The analyte, indicated in purple, is injected into the holes outside the MgF${}_{2}$ layer ring with diameter d2 = 1 μm and lattice pitch Λ2 = 6.050 μm. The thickness of gold and MgF${}_{2}$ layers are 50 nm and 40 nm, respectively [35]. For optimization, the following procedures are maintained step by step. First, we have tuned the inner air hole diameter and outer analyte hole diameter to gain high sensitivity response. After that, we have tuned the gold layer to optimize the gold layer thickness. Last, we have tuned the MgF${}_{2}$ layer to receive high sensitivity response and optimize the layer thickness. MgF${}_{2}$ is naturally birefringent, and it is used in the proposed PCF structure as a buffer which works for the betterment of sensitivity response. The RI of the background material is obtained using the Sellmeier equation [35]:

(1) $$n^{2}\left(\lambda ,T\right)=\left(6.90754\times 10^{-6}T+1.31552\right)+\frac{A}{B}+\frac{\lambda^{2}\left(0.548368\times 10^{-6}T+0.91316\right)}{\lambda^{2}-100}$$

where $A=\lambda^{2}\left(23.5835\times 10^{-6}T+0.788404\right)$; $B=\lambda^{2}-\left(0.584758\times 10^{-6}T+0.0110199\right)$; and effective RI, temperature, and wavelength are represented by *n*, *T*, and λ, respectively.

The dielectric constant of gold is defined by the Drude–Lorentz model [36]:

(2) $$\varepsilon_{g}=\varepsilon_{\alpha }-\frac{\omega_{d}^{2}}{\omega (\omega +jY_{d})}-\frac{\left(\Delta \varepsilon \times \Omega_{l}^{2}\right)}{\left(\omega^{2}-\Omega_{l}^{2}\right)-J\Gamma_{l}\omega }$$

The dielectric constant of gold, angular frequency, damping frequency, permittivity, plasmon frequency, and the weighting factor are represented by $\varepsilon_{g}$, ω, $Y_{d}$, $\varepsilon_{\alpha }$, $\omega_{d}$, and Δε, respectively. The constant values of spectral width, $\Gamma_{l}$; plasmon frequency, $\omega_{d}$; permitivity, $\varepsilon_{\alpha }$; damping frequency, $Y_{d}$; and resonant frequency, $\Omega_{l}^{2}$ are taken following the work in [36]. The RI of MgF${}_{2}$ has been chosen from reference [37].

A schematic diagram of the explored C-PCF based sensor is shown in Figure 1c. This setup included the polarization controller, optical tunable source (OTS), optical spectrum analyzer (OSA), and single-mode (SM) fiber.

## 3. Result Analysis and Discussion

**Figure 2:**
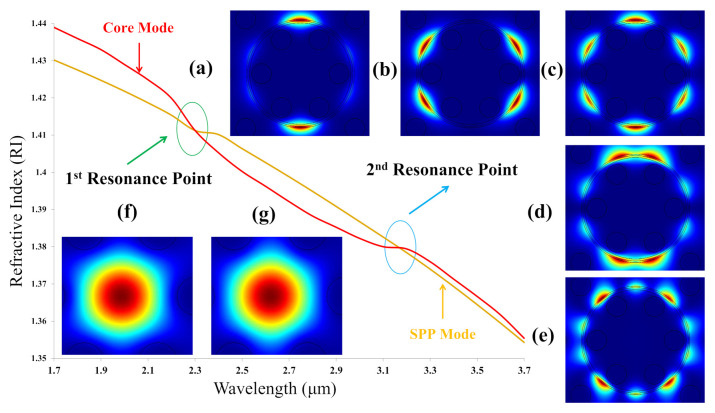
The representation resonance peak points for analyte 1.33 with SPP modes (**a**–**e**) first–fifth SPP modes; fundamental modes (**f**) X-polarization and (**g**) Y-polarization.

SPP is a specialized term in the metal surface field. An SPP mode is an electromagnetic excitation that exists at the metal surface. Figure 2 shows the resonance points of fundamental modes and SPP modes at 3 μm when RI of analyte equals to 1.33. Five SPP modes such as 1st, 2nd, 3rd, 4th, and 5th SPP modes are demonstrated in Figure 2a–e, sequentially. Additionally, two X and Y fundamental modes are displayed in Figure 2f–g.

### 3.1. Birefringence

**Figure 3:**
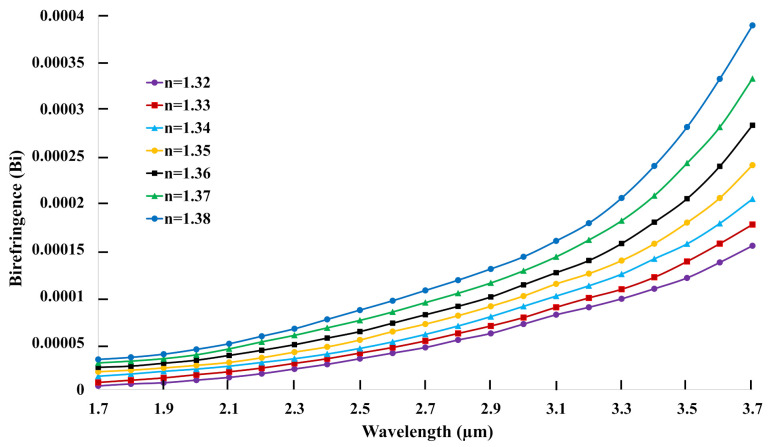
The birefringence curve of the proposed C-PCF structure versus wavelength.

Birefringence ($B_{i}$) is the RI difference between X polarization and Y polarization, which is measured using Equation (3) [38]:

(3) $$B_{i}=n_{x}-n_{y}$$

where $n_{x}$ and $n_{y}$ are the RI of fundamental mode at X and Y polarizations, respectively. In the whole operation, the wavelength varies from 1.7 to 3.7 μm with the increase of RI of analyte from 1.32 to 1.38. The birefringence variation of C-PCF structure with respect to wavelength is illustrated in Figure 3. The RI difference increases gradually with the increase of wavelength which is clearly noticeable in Figure 3. At the same time, there exists another correlation among the analytes, with higher RI of analytes exhibiting a higher birefringence. The highest birefringence of 3.9 × $10^{-4}$ is achieved when the RI of the analyte reaches 1.38. The sensing signals can easily be separated for higher birefringence, which makes the explored C-PCF an exalted candidate for sensing applications.

### 3.2. Coupling Length

Coupling length is another important parameter in measuring the performance of sensor, which is related to birefringence. After measuring the birefringence, coupling length can easily be calculated, as it has a inverse relationship with birefringence, using Equation (4) [12]:

(4) $$L_{c}\;\left(\mu m\right)=\frac{\lambda }{2\times B_{i}}$$

**Figure 4:**
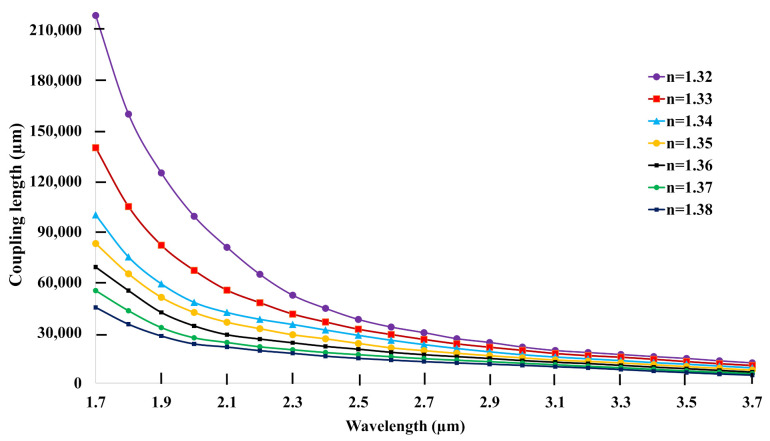
The coupling length of the proposed C−PCF versus wavelength.

The coupling length ($L_{c}$ in μm) of the explored C-PCF for analytes 1.32 to 1.38 is shown in Figure 4. Following the inverse relationship, the coupling length decreases with the increase of wavelength. Additionally, the downward curve decreases very rapidly from 1.7 μm to 2.4 μm, but, after that, it decreases very slowly. At the same time, there exists another correlation among the analytes: a higher RI of analyte exhibits a shorter coupling length. This happened because of lower birefringence and higher loss peak response of lower analytes. The higher coupling length ensures long filling length, long filling time, and large volumes of analytes [39]. The explored C-PCF exhibits 60 mm and 30 mm coupling lengths at 1st and 2nd resonance points, respectively, for analyte n = 1.32.

### 3.3. Output Power Spectrum

**Figure 5:**
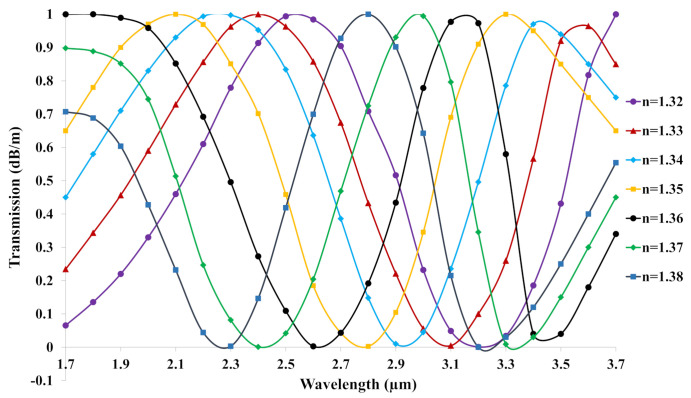
The transmission spectrum variations in dB/m scale of the proposed C−PCF versus wavelength.

The output power spectrum is related to birefringence and can be defined by its coupling length. The power spectrum ensures fiber efficiency, which is calculated using the Equation (5) [12]:

(5) $$Power_{out}\;\left(\mathrm{dB}/m\right)=sin^{2}\left(\left(B_{i}\pi l\right)/\lambda \right)$$

where *l* is the acting fiber length. Figure 5. displays the transmission spectrum of the explored C-PCF for RI = 1.32–1.38. The power ranged from 1 to 0 following the sinusoidal curve. The higher RI of analyte has a denser curve than that of the lower RI of analyte. As a result, the output spectrum of analyte 1.38 was the most lagged, while RI of analyte 1.32 was the most forward.

### 3.4. Transmission Spectrum

The transmission spectrum is used to calculate transmittance, which is a significant parameter in measuring the performance of the PCF. Transmittance is evaluated using Equation (6) [12]:

(6) $$T\;\left(\mathrm{dB}\right)=10log_{10}\left(P_{out}/P_{in}\right)$$

**Figure 6:**
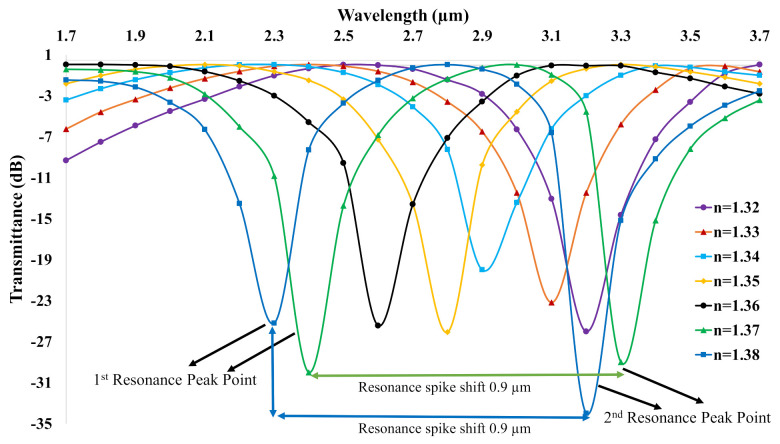
Transmittance variations in dB scale of the proposed C−PCF versus wavelength.

Here, *T* is the transmittance in dB, and $P_{out}$ and $P_{in}$ are the maximum output and input powers, respectively. The transmittance spectrum is shown in Figure 6 for RI = 1.32–1.38. The maximum transmittance of −34 dB is achieved for RI = 1.38. The other calculated transmittance profiles are −26 dB, −23 dB, −20 dB, −26 dB, −25 dB, and −30 dB for RI = 1.32–1.37, respectively. The higher RI of analyte had a denser curve than that of the lower RI analyte with a sharp downward peak. As a result, higher RI of analytes 1.38 and 1.37 exhibited two sharp downward peaks, respectively, while the rest exhibited one sharp downward peak. Moreover, the transmittance profile of analyte with n = 1.38 was the most lagged, while n = 1.32 was the most forward.

### 3.5. Loss Spectrum

The loss spectrum of light confinement (CL) is a crucial parameter to calculate the sensor response, which is calculated using Equation (7) [40]:

(7) $$\alpha \;\left(\mathrm{dB}/m\right)=8.686\times \left(2\pi /\lambda \right)\times Im\left[n_{eff}\right]\times 10^{6}$$

where $Im[n_{eff}]$ stands for the imaginary part of the RI and proportional to loss profile. Besides, CL is inversely proportional to the operating wavelength.

MgF${}_{2}$ is naturally birefringent, an ideal material that applied in the models as a buffer for the betterment of sensitivity. The wavelength sensitivity is measured by taking the value of the RI difference and peak wavelength variation from the loss curve using the Equation (8) [41,42]:

(8) $$S_{w}\;\left(\mathrm{nm}/\mathrm{RIU}\right)=\Delta \lambda_{p}/\Delta n$$

where Δn and $\Delta \lambda_{p}$ are the effective RI and the peak wavelength difference, respectively. The wavelength sensitivity is proportional to the peak wavelength variation and inversely proportional to the effective RI difference. Therefore, the entire performance of the C-PCF structure can easily be measured based on resolution profile, which is calculated using the following equation [41]:

(9) $$R\;\left(\mathrm{RIU}\right)=\left(\lambda_{min}\times \Delta n\right)/\lambda_{p}$$

where Δn is the effective RI variation, $\lambda_{p}$ is the peak wavelength difference, and $\lambda_{min}$ is the minimum spectral resolution.

**Figure 7:**
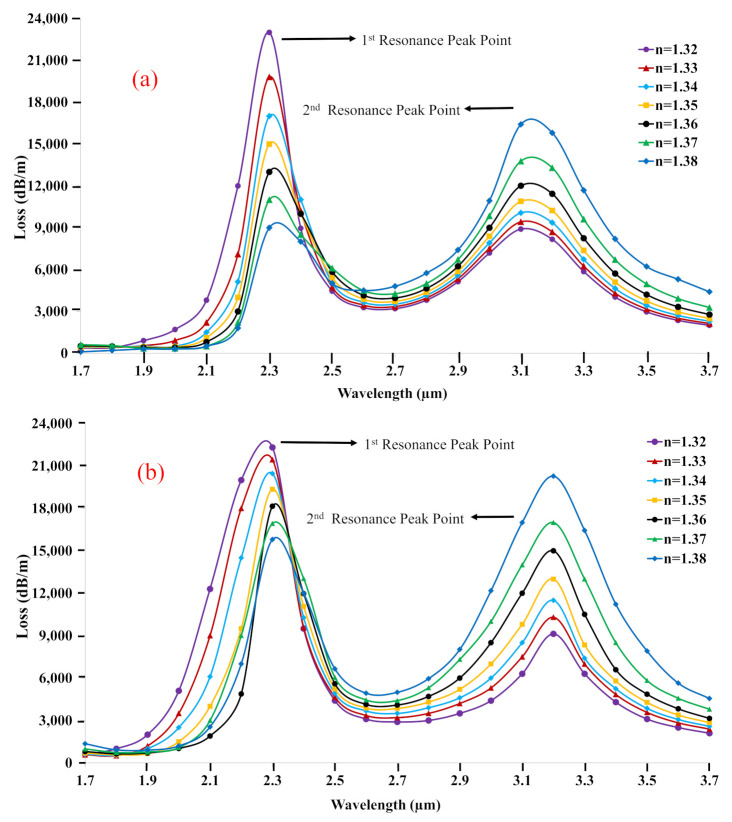
The Confine-loss profile of the proposed C−PCF for (**a**) X- (**b**) Y-polarization versus wavelength.

Figure 7a shows the CL spectrum of the proposed C-PCF structure for X polarization. Only two peaks are chosen to calculate the confinement loss spectrum. There exists a different relationship in the first peak and second peak among the RI analytes. In the first peak, the amplitude of the loss curve increases with the decrease of the RI of analytes. On the other hand, the amplitude of the loss curve increases with the increase of the RI of analytes in the 2nd peak. The wavelength sensitivity of 27,958.49, 27,935.93, 27,916.89, 27,900.32, 27,883.93, 27,850.92, and 27,797.29 nm/RIU were obtained for RI = 1.32–1.38, respectively. However, the calculated resolution profiles were 3.57673 × $10^{-5}$, 3.57962 × $10^{-5}$, 3.58206 × $10^{-5}$, 3.58419 × $10^{-5}$, 3.58629 × $10^{-5}$, 3.58411 × $10^{-5}$, and 3.59747 × $10^{-5}$ RIU, correspondingly.

In Figure 7b, for Y polarization there exists the same relationship among the RI analytes like X polarization, which means the amplitude of the loss curve increases with the decrease of the RI analytes in the 1st peak, and the amplitude of the loss curve increases with the increase of the RI analytes in the 2nd peak. The sensitivity of 27,020.16, 27,049.15, 27,085.31, 27,128.00, 27,177.32, 27,226.43, and 27,237.45 nm/RIU were gained for RI analyte 1.32–1.38, respectively. Moreover, the measured resolutions were 3.70094 × $10^{-5}$, 3.69697 × $10^{-5}$, 3.69204 × $10^{-5}$, 3.68623 × $10^{-5}$, 3.67954 × $10^{-5}$, 3.6729 × $10^{-5}$, and 3.67142 × $10^{-5}$ RIU, correspondingly.

**Figure 8:**
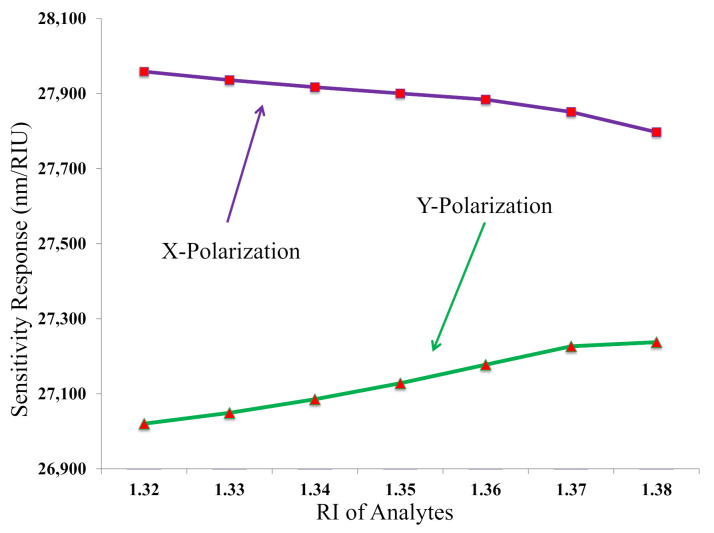
The sensitivity response with respect to RI of analytes for X- and Y-polarizations.

Figure 8 describes the wavelength sensitivity with respect to the RI of analyte for both polarizations. The sensitivity increases or decreases depending on the peak wavelength variations and RI variations. It is clear from Equation (9) that the sensitivity increases if the peak wavelength variation increases or the RI analyte variation decreases, and vice versa. The utmost sensitivity of 27,958.49 nm/RIU was gained for X polarization at RI = 1.32, where the other calculated sensitivity were 27,935.93, 27,916.89, 27,900.32, 27,883.93, 27,850.92, and 27,797.29 nm/RIU for RI = 1.33–1.38, respectively. In Figure 8, the sensitivity decreases with the increase of RI of analyte, and, as a result, the highest sensitivity is gained at the lowest RI of analyte for the X polarization. This happens because the RI difference increases with the increase of RI of analyte. On the other hand, the sensitivity increases with the increase of RI of analyte. As a result, the maximum sensitivity of 27,237.45 nm/RIU was gained for the Y polarization at the highest RI = 1.38. The other sensitivity of 27,020.16, 27,049.15, 27,085.31, 27,128.00, 27,177.32, and 27,226.43 nm/RIU were obtained for RI = 1.32–1.37, respectively. This occurs because the RI difference decrease with the increase of RI of analyte for the Y polarization. Table 1 shows a comparative analysis of the sensitivity and resolution between the existing PCF sensors and C-PCF sensor. From Table 1, it is clear that the proposed sensor has achieved a better sensitivity and resolution compared to existing PCF sensors.

## 4. Fabrication Possibility and Application Area

The fabrication process of the microstructure-based PCF sensor is more complex compared to the conventional optical fiber. Some fabrication methods like wheel polishing method (WPM) [45], sol–gel [46], chemical vapor deposition (CVD) [47], atomic layer deposition (ALD) [48], stack-and-drilling, stack-and-draw [49,50], 3D printing, injection modeling, capillary stacking, and extrusion techniques [51] are very well known. Applying the drilling technique, a computer-controlled mill has been used to drill air holes in the solid rode and solid tube, and draws them to the inner and outer air structure fibers, respectively [52]. Using the chemical vapor deposition (CVD) method, gold has been deposited at the outer side of the microfiber [53,54]. In the same way, MgF${}_{2}$ has been deposited at the inner side of the fiber. For example, a Au–MgF${}_{2}$-coated nanostructure is briefly described experimentally in [55] by varying the layer size and parameters. Additionally in the case of fabrication, some SPR-based optical fiber sensors with Ag–Tio${}_{2}$-coated [56], Au-coated [57,58,59], and MgF${}_{2}$/TiO${}_{2}$-coated optical filter [60] are experimentally demonstrated. In fabrication summary, a long period of operating wavelength makes the proposed sensor more attractive for both sensing and telecommunications applications owing to their low losses profile, compact sizes, ease of fabrication, and low levels of back-reflection [61,62].

In the application area, SPR-based sensors can be applied for bioimaging, medical diagnostics, organic chemical sensing, liquid sensing, disease detection, gas sensing, glucose monitoring in urine, tuberculosis detection, pregnancy testing, environment monitoring, etc. [63]. The proposed sensor can be applied as a biosensor because the operating analyte range is 1.32 to 1.38. A large number of biological analytes’ refractive indexes lie in the range of 1.32 to 1.38, for example, tuberculosis cells sensing (1.345–1.349) [64], pregnancy testing (1.335–1.343) [35], cancer cell detection (1.36–1.38) [65], alcohol sensing (1.333–1.3611), different blood components sensing (1.33–1.40) [66], etc. Therefore, the large range of analytes and high sensing performance of the proposed sensor make it an efficient candidate in the vast numbers of SPR-based biosensor application areas.

## 5. Conclusions

A twin resonance peak-based RI sensor is explored and numerically demonstrated using the FEM method. To gain maximum sensing performance of 27,958.49 nm/RIU, a nano-film gold layer and a MgF${}_{2}$ layer were used as a plasmonic material. Besides, high birefringence, resolution, and transmittance of 3.9 × $10^{-4}$, 3.70094 × $10^{-5}$ RIU, and −34 dB were obtained, respectively. In conclusion, the displayed C-PCF will be a potential candidate in twin resonance peak-based sensing areas for its excellent sensing performance.

## Figures and Tables

**Table 1:** Performance comparison between previously published paper and C-PCF structure.

Structures	$S_{w}$ (nm/RIU)	*R* (RIU)	Publication Year	Reference
SPR based D-shaped sensor	7700	1.30 × $10^{-5}$	2017	[29]
Dual-core PCF based RI sensor	9000	1.10 × $10^{-5}$	2018	[43]
PCF based D-shaped RI sensor	10,493	9.53 × $10^{-6}$	2017	[30]
No-core multimode SPR sensor	11,792	2.04 × $10^{-5}$	2019	[44]
Selectively coated PCF sensor	11,000	9.10 × $10^{-6}$	2018	[31]
D-shaped SPR based PCF sensor	11,500	8.70 × $10^{-6}$	2020	[32]
Gold-and MgF2-Coated RI sensor	27,959	3.70 × $10^{-5}$	-	Proposed Work

## Data Availability

Not applicable.
